# Coping, meaning in life, and quality of life during ongoing conflict: insights from Israeli populations

**DOI:** 10.1186/s13584-024-00665-1

**Published:** 2025-01-08

**Authors:** Orly Sarid, Liat Hamama, Yaira Hamama-Raz

**Affiliations:** 1 The Spitzer Department of Social Work, Ben-Gurion University of the Negev, Beer Sheva, 84105 Israel; 2 The Bob Shapell School of Social Work, Tel Aviv University, Tel Aviv, Israel; 3 School of Social Work, Yaira Hamama-Raz, Ariel University, Ariel, Israel

**Keywords:** Traumatic events, Health-related quality of life, Meaning in life, Coping strategies, Self-mastery, Evacuation

## Abstract

**Background:**

Conducted in May 2024, this study examines the well-being of Israeli evacuees and non-evacuees from conflict zones. We assess health-related quality of life (HRQoL), meaning in life (MIL), coping strategies, psychological symptoms, and self-mastery. Aims include exploring effects of trauma and socio-demographics on HRQoL and MIL, analyzing mediating roles of psychological symptoms and coping, and evaluating if evacuation status moderates these relationships during ongoing conflict.

**Methods:**

In May 2024, seven months post–October 7th attacks, we conducted a cross-sectional study with 366 participants (221 evacuated, 145 non-evacuated) via a survey company. We assessed HRQoL (SF-12), MIL (MLQ), psychological symptoms (PHQ-4), self-related and other-related coping strategies (Brief COPE), and self-mastery (Self-Mastery Scale) through self-reported measures. Path and moderated mediation analyses evaluated relationships among socio-demographics, psychological symptoms, coping variables, HRQoL, and MIL.

**Results:**

Except for self-mastery, northern and southern evacuees showed no significant differences and were combined into one group. Path analysis revealed significant associations between traumatic life events, HRQoL, and MIL. Traumatic events were negatively associated with the physical component summary (PCS) of HRQoL and positively with anxiety, depression, and coping (self and others- problem-solving). Depression negatively related to PCS, mental component summary (MCS), and MIL, while coping (self and others) was positively associated with MIL. Moderated mediation analysis showed evacuated participants had higher dysfunctional coping, whereas non-evacuated participants demonstrated a stronger positive relationship between anxiety and the search for meaning.

**Conclusion:**

Despite regional differences, evacuees exhibited similar psychological responses, likely due to the shared experience of displacement. Traumatic events negatively impacted their HRQoL and MIL. Adaptive coping strategies—self-related and problem-focused coping through helping others—played significant roles in mitigating these effects. The theoretical frameworks of Conservation of Resources (COR) theory, Taylor’s “tend and befriend” model, and Frankl’s existential framework provided a basis for explaining these findings.

**Supplementary Information:**

The online version contains supplementary material available at 10.1186/s13584-024-00665-1.

On October 7, 2023, an attack by Hamas precipitated a massacre, numerous abductions, the outbreak of war, and the evacuation of approximately 126,000 people [1]. This study, focused on individuals from the southern and northern regions of Israel who were evacuated due to the conflict, as well as those who remained in their homes further inland with less direct exposure. Both evacuees and non-evacuees were surveyed to assess various aspects of their well-being, including quality of life, meaning in life, coping strategies, and psychological symptoms.

## Background

Health policymakers face significant challenges in ensuring the well-being of people in conflict zones, where increased health risks and disrupted protective factors are prevalent [2].

Traumatic life events significantly influence individuals’ physical and mental health, affecting their health-related quality of life (HRQOL) and sense of meaning in life (MIL). Guided by Taylor’s bio-psycho-social framework [3] which conceptualizes health outcomes as processes linking environmental factors, stress responses, and coping resources—we explored both direct and indirect paths from traumatic life events and socio-demographic variables to HRQOL and MIL outcomes.

HRQOL is defined as an individual’s functioning and perceived well-being in physical and mental health domains [4]. MIL, on the other hand, is characterized by beliefs, values, and goals that provide purpose and coherence [5, 6]. Frankl [7] emphasized that life can hold significant meaning even in dire circumstances, highlighting the role of inherent human significance in adapting to and overcoming traumatic experiences [8–10].

Building on Taylor’s model, we examined the direct effects of traumatic life events and socio-demographic factors on HRQOL and MIL. Additionally, we investigated indirect effects mediated by symptoms, coping strategies, and self-mastery, drawing on Hobfoll’s conservation of resources theory (COR) [11]. According to Hobfoll, individuals strive to obtain, retain, and protect their resources, and the loss or threat of loss of these resources can lead to stress and adverse health outcomes.

In this study, we assessed the health outcomes of evacuees from both the northern and southern regions of Israel, as well as individuals who were not evacuated. Despite the different contexts of evacuation, each stemming from distinct traumatic events—we hypothesize that coping strategies, psychological symptoms, and health outcomes may be consistent across the two groups of evacuees. In other words, the experience of evacuation may overshadow other differences, influencing all aspects of their experiences.

Based on Taylor’s model we explored literature looking for direct paths from traumatic life events and socio-demographic variables to HRQoL and MIL outcomes. We then explored indirect paths through symptoms, coping strategies, and self-mastery using Hobfoll’s conceptualization.

### Relationships between traumatic life events, socio-demographic variables with HRQoL and MIL

The theoretical model of COR [11], provides valuable insight into the impact of displacement, separation from social networks, and the traumatic events associated with wartime on mental health. According to the COR model, individuals strive to obtain, retain, and protect their resources, with stress occurring when resources are threatened or lost [11]. This model has been applied to examine responses to traumatic and high-stress situations, especially in conflict and disaster contexts [12–14]. The COR framework emphasizes that the loss of personal, social, or material resources significantly affects mental health and well-being [15]. Previous research indicated that traumatic life events including violence, abuse, and life-threatening diseases have been linked to lower HRQoL, particularly among women [16].

Socioeconomic status (SES) significantly impacts physical health, though its effect on psychological health was less evident [17]. However, a recent study demonstrated that when individuals have a more positive subjective appraisal of their SES, their physical and mental HRQoL also tends to increase [18]. Additionally, low educational attainment has been associated with lower HRQoL [19]. Regarding gender, women in nurturing roles often experience a decrease in HRQoL, as they bear a disproportionate burden of caregiving responsibilities in the domestic sphere compared to their male counterparts [20].

Concerning MIL, SES (including subjective SES), was found to be positively correlated with meaning in life [21–23]. Economic resources may enhance purpose, significance, and coherence, which are vital components of meaning [19]. Women were found to have higher levels of the presence of MIL and a higher tendency to search for MIL than men [6].

### Relationships between socio-demographic variables and the mediators’ variables (psychological indicators, coping strategies, and self-mastery)

Socio-demographic factors, such as age, place of residence, marital status, and financial status, are linked to psychological symptoms such as anxiety and depression during disasters [25, 26]. For example, Ukrainian adults during the Russian invasion showed higher depression levels if they were older, lived in rural areas, were unmarried, or had lower financial status [25]. Women college students in disaster-prone areas in Indonesia exhibited higher anxiety than men [26].

In the context of coping strategies, scholars define *coping* as conscious cognitive and behavioral processes used to manage stressful physical and psychological events [27–29]. In this study, we focused on the Carver COPE [27] scale as a method to monitor individuals’ conscious coping strategies following trauma, rather than utilizing support scales that measure perceived social support. This approach emphasizes the importance of active engagement in coping processes, as opposed to passive reliance on perceived support. Common coping strategies include emotion-focused coping, which aims to reduce emotional burdens through activities such as actively reaching out to others for social and instrumental support, humor, acceptance, and religious rituals. Another strategy is problem-focused coping, which addresses stress through actions like active coping, seeking informational support, planning, and positive reframing. Lastly, dysfunctional coping involves maladaptive behaviors such as avoidance, self-blame, denial, and substance use, including smoking, drugs, and alcohol [30]. A study among internally displaced individuals and host communities in war-affected regions of Nigeria found that being single, living in camps, and aged 18–29 predicted the use of emotion-focused and dysfunctional coping strategies [31]. Interestingly, individuals aged 18–29 and over 50 also employed problem-focused coping strategies, suggesting that younger adults may utilize a broader range of coping mechanisms.

In the current study, we also examine coping strategies that involve helping others. We extended Taylor’s [32] original “tend and befriend” concept to explore the role of providing social support through coping processes. Coping strategies that involve aiding others not only mitigate stress effects but also enable individuals to better understand and articulate the support they provide, touching upon underexplored intrapersonal aspects of coping [33, 34]. Coping for others involves behaviors, emotions, and cognitive expressions aimed at helping others, such as problem-solving, giving advice, or offering humor and emotional support. The literature lacks comprehensive measures for these strategies; therefore, we adapted Carver’s [35] questionnaire to include coping related to helping others. According to Frankl [7], individuals who helped others in concentration camps were better able to cope with their helplessness, maintained resilience, and found meaning. Prioritizing others’ needs can cultivate a sense of purpose, even in adversity, suggesting an altruistic pathway that benefits the helper by fostering social connections and enhancing well-being [36, 37]. It is important to note that coping related to helping others is a strategy for managing ongoing stress, with outcomes that can be adaptive or maladaptive for the provider depending on the context [38]. Consequently, its impact on the provider’s mental health is not predetermined. Finally, regarding the link between socio-demographic variables and *self-mastery*—defined as an individual’s perceived control over life events [39] and known to enhance coping with stress [40]—a previous study found higher self-mastery levels among men, as well as individuals with higher education, higher income, and better health status [41].

### Relationships between mediators’ variables with HRQoL and MIL

Previous research has demonstrated a negative association between psychological symptoms of anxiety and depression with mental and physical quality of life (QoL) [42]. Additionally, these psychological symptoms were linked to a diminished sense of purpose in life [43].

As for self-coping strategies, dysfunctional coping was linked to decreased QoL, while problem- and emotion-focused strategies improved QoL over time during the COVID-19 pandemic [44]. Similarly, MIL was strongly correlated with problem- solving and emotion-focused adaptive coping strategies in the face of daily stressors and severe trauma [45]. Specifically, MIL has been linked to problem-focused coping mechanisms, such as positive reinterpretation, proactive planning, and self-efficacy, and to a lesser extent, emotion-focused behaviors [45]. The concept of self-mastery is discussed in the context of displacement and evacuation. This is particularly relevant where stressors from both material and interpersonal sources disrupt daily routines and contribute to poorer mental health [46, 47]. Hou and colleagues [48] identified two types of daily routines: primary and secondary. Primary routines involve behaviours necessary for maintaining a livelihood, such as hygiene, sleep, eating, and home maintenance. In contrast, secondary routines are optional and depend on motivation and preferences, including activities like exercising, leisure, social activities, and employment. Both routines are crucial for maintaining a sense of normalcy and control over one’s life. In the context of war, displacement exacerbates the irregularity of daily routines and reduces the sense of predictability and coping flexibility, which, in turn, leads to deteriorating mental health over time [46]. High self-mastery can potentially mediate the negative effects of disrupted routines by promoting adaptive coping mechanisms. It enables individuals to maintain feelings of autonomy and self-efficacy, fostering innovative problem-solving strategies [49]. The current study aims to highlight the role of self-mastery—the ability to cope with the challenges of displacement, and its effects on health and the finding of MIL.

Given the context, the objectives of the present study are as follows:

1. To compare evacuees from the northern and southern regions of Israel in terms of HRQoL, MIL, psychological indicators, self-coping strategies, self-mastery, and coping strategies related to helping others.
2. To investigate the direct relationships between the independent variables (traumatic life events and socio-demographic background) and the dependent variables (HRQoL and MIL).
3. To analyze the direct relationships between the independent variables and the mediating variables, including psychological indicators, self-coping strategies, self-mastery, and coping strategies related to helping others.
4. To evaluate the relationships between the mediating variables and the dependent variables (HRQoL and MIL). We hypothesize that psychological indicators (anxiety and depression) will negatively correlate with both HRQoL and MIL. We further hypothesize that individuals who utilize adaptive coping strategies (self/others problem- and emotion-focused coping) will report higher HRQoL and greater MIL.
5. To examine a mediation model assessing the role of mediators (psychological symptoms, self-coping strategies, self-mastery, and coping strategies related to helping others) on the relationship between the independent variables (socio-demographic background and traumatic life events) and the dependent variables (HRQoL and MIL). We hypothesize that group status (evacuee versus non-evacuee) will serve as a moderator in the mediation model.

## Methods

This cross-sectional study was conducted in Israel in May 2024, seven months after the October 7th attack and the beginning of the Swords of Iron War. The research protocol was approved by the three principal researchers’ ethics committees. Participants were recruited via iPanel, Israel’s leading online research platform, which follows ESOMAR’s international guidelines and manages a panel of about 100,000 members.

The inclusion criteria for the sample were as follows: Participants had to provide informed consent, be at least 21 years old, fluent in Hebrew and have been evacuated from their homes within the first month after October 7, 2023. Exclusion criteria included pregnant women, those not proficient in Hebrew, returned hostages, and individuals who declined participation. All participants were fully informed about the study’s aims and consented before participating.

### Participants

The sample size for the moderated-mediation hypothesis was calculated via two separate power analyses. The mediation aspect was assessed using a Monte Carlo power simulation through the mc_power_med app [50], indicating that a minimum of 300 participants would be required to achieve 80% power (1-β), based on medium effect sizes for each model path (*r* = .3), and an alpha level of 0.05. For the moderation component, G*Power 3.1.9^51^ was employed, which estimated that 120 participants would be sufficient to detect a medium effect size (*f*^2^ = 0.15) with at least 80% power (1-β) and an alpha of 0.05. The study’s actual sample size of 360 participants surpassed both estimates, thereby ensuring ample statistical power for both components.

The study involved 366 participants: 221 individuals evacuated from war zones in southern and northern Israel, and 145 individuals who were not evacuated due to their greater distance from the conflict borders in the northern and southern regions (a control group). Participants’ ages ranged from 19 to 82 years (*M* = 40.93, *SD* = 13.50). Most participants were female (62.8%, *n* = 230), married or in a committed relationship (60.7%, *n* = 222), and reported an average or below-average income (66.9%, *n* = 245). About half had tertiary education (51.1%, *n* = 187). No significant sociodemographic differences were found between evacuees and non-evacuees, except for the fact that there were more women in the evacuee group (69.7% vs. 52.4%).

### Measures

Participants completed a series of standardized self-report questionnaires, previously validated for Israeli populations and showing strong psychometric properties. The measures are grouped according to their role in the study:

### Independent variables

*Sociodemographic data* included information on participants’ gender, age, marital status, education, income, region of residence (north or south), and whether they were evacuated from their homes.

*Traumatic life events* were assessed using the List of Threatening Experiences Questionnaire (LTE-Q [52]), which evaluates 12 major adverse events from the past two months, such as job loss or serious illness, using a yes/no format. Among our participants, 35.5% (*n* = 130) reported no traumatic events, 38.3% (*n* = 140) reported one to two events, and 26.2% (*n* = 96) reported three and more events. The most frequently reported events were the death of a close family friend or relative (27%), a serious illness or injury of a close relative (24.3%), unemployment or prolonged job-seeking lasting over a month (21.9%). The original questionnaire demonstrated Cronbach’s alpha of .84^52^, while the current study yielded an acceptable alpha of 0.70.

### Mediating variables

*Depression and anxiety* were assessed using the Patient Health Questionnaire 4-item (PHQ-4) [53], which includes a 2-item depression scale (e.g., “Feeling down, depressed, or hopeless”) and a 2-item anxiety scale (e.g., “Feeling nervous, anxious, or on edge”). Participants rated how often they were bothered by certain problems over the past two weeks on a 4-point scale from 0 (“not at all”) to 3 (“nearly every day”). The original PHQ-4 had Cronbach’s alphas of 0.85 for depression and 0.81 for anxiety [53]. In this study, the alphas were 0.86 for depression and 0.84 for anxiety.

*Coping strategies (self)* were measured using the Brief COPE questionnaire [35], which assesses coping using 14 subscales (each subscale has 2 items) divided into three overarching coping styles: emotion-focused (12 items; e.g., “I’ve been getting emotional support from others”), problem-focused (8 items; e.g., “I’ve been thinking hard about what steps to take”), and dysfunctional strategies (8 items; e.g., “I’ve been giving up trying to deal with it”) [27]. Each item was rated on a 4-point scale from 1 (“I haven’t been doing this at all”) to 4 (“I’ve been doing this a lot”), with higher scores indicating greater degree to which the respondent has been engaging in that coping style. In this study, Cronbach’s alphas were 0.71 for emotion-focused, 0.84 for problem-focused, and 0.70 for dysfunctional strategies.

*Coping strategies (other)* were measured by adapting Brief COPE [35] to assess coping through aiding others. Items from the original subscales were reworded to reflect helping behaviors, capturing how individuals cope with stress by supporting others rather than focusing solely on intra-personal strategies (e.g., “I taught someone how to take action to improve the situation”, “I supported someone who told me they refused to believe it happened”, “I encouraged someone to use alcohol or other substances to improve their mood”).

A confirmatory factor analysis (CFA) was conducted with the study participants. The analysis revealed poor fit indices [χ^2^(347) = 2266.57, χ^2^/*df* = 6.53, CFI = 0.69, RMSEA = 0.12, SRMR = 0.11], with 7 items (five from dysfunctional coping and two from emotion-focused coping) showing low loadings. As a result, the decision was made to focus solely on the problem-focused coping factor. The CFA for the problem-focused coping items indicated a poor to excellent fit [χ^2^(20) = 143.60, χ^2^/*df* = 7.18, CFI = 0.93, RMSEA = 0.13, SRMR = 0.06]. An examination of modification indices suggested adding a correlation between the error terms of items 12 (“I taught someone to view the situation in a different light, making it appear more positive to them) and 17 (“I’ve been looking for something good in what is happening”) to improve the fit. This adjustment significantly improved the fit indices (Δχ² = 127.57, Δ*df* = 9, *p* < .001), supporting a three-factor structure with acceptable to excellent fit indices [χ^2^(19) = 56.09, χ^2^/*df* = 2.95, CFI = 0.98, RMSEA = .07, SRMR = .03]. The internal consistency of the problem-focused coping (other) factor was excellent α = .92.

*Self-mastery* was assessed using the Self-Mastery Scale [39], which comprises seven statements that measure the extent to which individuals perceive their life outcomes as being under their control rather than determined by fate (e.g., “I have little control over the things that happen to me”). Participants responded using a 4-point Likert scale ranging from 1 (strongly disagree) to 4 (strongly agree), with higher scores indicating a weaker sense of personal mastery. In this study, Cronbach’s alpha was 0.77.

### Dependent variables

#### Health-related quality of life

(HRQoL) was assessed using the Short Form-12 Health Survey (SF-12) [54], a tool designed to evaluate the physical and mental components of quality of life. It consists of 12 questions across eight health domains: physical functioning, role limitations due to physical health, bodily pain, general health perceptions, vitality, social functioning, role limitations due to emotional problems, and mental health. Responses are combined into two subscales: the Physical Component Summary (PCS) and the Mental Component Summary (MCS), each ranging from 0 (worst) to 100 (best). Cronbach’s alphas were 0.80 for the PCS and 0.81 for the MCS.

#### Meaning in life

(MIL) was assessed using the Meaning in Life Questionnaire (MLQ) [5], which includes two subscales: the presence of meaning (MIL-P) and the search for meaning (MIL-S). The MIL-P measures how meaningful a person perceives their life to be (5 items: e.g., “I understand my life’s meaning”), while the MIL-S assesses their motivation to find or deepen life’s meaning (5 items; e.g., “I am searching for meaning in my life”). Each item is rated on a 7-point scale from 1 (Absolutely untrue) to 7 (Absolutely true), with higher scores indicating greater meaning in life. In the original scale, Cronbach’s alphas were 0.87 for MIL-S and 0.86 for MIL-P. In this study, they were 0.87 for MIL-S and 0.91 for MIL-P.

### Statistical analysis

Before conducting the analyses, the dataset was examined for outliers and assessed for normality. No outliers were identified [55], and all variables exhibited normal skewness and kurtosis values [56]. For the study’s first aim, we analyzed whether evacuees from the northern and southern regions of Israel differed in their experiences and responses. Independent samples t-tests were used for continuous variables, and chi-square (χ²) tests of independence were performed for categorical variables.

Based on Taylor’s model bivariate analyses were subsequently performed to investigate the relationships between background variables, mediators, and dependent variables. Pearson’s correlations were utilized for continuous variables, while point-biserial correlations were employed for associations involving dichotomous and continuous variables.

To address study aims 2 through 5, path analysis was conducted with paths specified according to the results of the bivariate analyses. Independent variables included gender, income, and traumatic life events; mediators comprised anxiety, depression, coping measures (self and other), and self-mastery; dependent variables encompassed HRQoL and MIL measures; and covariates included age, family status, education, and evacuation. Model estimation was performed using the maximum likelihood method, and model fit was evaluated using several goodness-of-fit-indices [57]: the χ^2^/df index, which is deemed acceptable if less than 5 and excellent if between 1 and 3; the Comparative Fit Index (CFI), with values above 0.90 considered adequate and above 0.95 considered excellent; the Root Mean Square Error of Approximation (RMSEA), with values below 0.08 indicating an adequate fit and below 0.06 indicating an excellent fit; Standardized Root Mean Squared Residual (SRMR), with values below 0.10 considered adequate and below 0.08 considered excellent. Non-significant paths were excluded to achieve the most parsimonious model [58], followed by a comparison between the initial and parsimonious models. A modification indices test was subsequently conducted to determine whether additional paths could be incorporated to improve the fit of the parsimonious model, followed by a comparison between the parsimonious and modified models. Model comparisons were conducted using the Expected Cross-Validation Index (ECVI), Akaike’s Information Criterion (AIC), and Bayes Information Criterion (BIC), with lower values indicative of better fit [59]. To evaluate the significance of the indirect effects, confidence intervals (CI) were estimated for each indirect effect based on 5,000 bootstrap samples [60]. An indirect effect was considered significant if the CI did not include zero [61]. A multiple group path analysis was also conducted based on evacuation, comparing a fully unconstrained model with no equality constraints, to a constrained model with all coefficients held constant across groups. A significant χ [2] square difference test suggests evidence of non-invariant parameters [62], and each path was tested for invariance. Additionally, moderated mediation analyses were conducted exclusively for relevant indirect effects, specifically, those comprising at least one path moderated by evacuation during war.

The statistical tests were performed using IBM SPSS Statistics version 29. Path analysis was conducted using IBM SPSS Amos version 24, employing the “Specific indirect effects” [63] to analyze the specific indirect effects; “Multigroup Analysis” plugin [64] to examine multiple group path analysis, including path comparisons by evacuation during war; and “MyModMed” estimand [56] for moderated-mediation analyses. An alpha level of 0.05 (two-tailed) was set for all statistical tests.

## Results

Regarding the first aim of the study, Table 1 presents the background characteristics, mediators, and dependent variables of evacuees, stratified by their residential area prior to evacuation. Notably, except for self-mastery, all comparisons between these groups across these variables yielded non-significant results. Therefore, we combined them into a single evacuee group.

**Table 1:** Background variables, mediators, and dependent variables of evacuees by residential area before evacuation

	North		South			
Characteristic	(*n =* 102)		(*n =* 119)			
**Background**	M	SD	M	SD	t	d
Age	40.54	12.38	41.40	13.95	-0.48	-0.07
Traumatic life events	1.01	0.88	1.08	0.92	-0.54	-0.07
Income	2.83	1.28	2.84	1.32	-0.06	-0.01
	***n***	**%**	***n***	**%**	**χ** ^**2**^	
Gender					0.32	
Male	29	28.4	38	31.9		
Female	73	71.6	81	68.1		
Family status					0.16	
Married or in a relationship	58	56.9	78	66.1		
Other	44	43.1	40	33.9		
Education					0.02	
Non-academic	49	48.0	56	47.1		
Academic	53	52.0	63	52.9		
***Questionnaire***	***M***	***SD***	***M***	***SD***	***t***	***d***
Anxiety	2.66	1.81	2.44	1.87	0.89	0.12
Depression	2.63	1.94	2.39	1.87	0.94	0.13
Problem-focused coping - Self	18.98	4.98	20.22	5.80	-1.69	-0.23
Emotion-focused coping - Self	26.00	5.36	26.94	6.49	-1.18	-0.16
Dysfunctional coping - Self	15.77	4.39	15.70	4.24	0.13	0.02
Self-mastery	2.22	0.59	2.07	0.49	2.09^*^	0.28
Problem-focused coping - Other	17.47	5.84	18.89	6.88	-1.64	-0.22
PCS	50.13	9.51	48.62	9.76	1.16	0.16
MCS	35.42	12.59	37.81	11.07	-1.50	-0.20
Search for meaning	23.15	6.97	23.05	6.88	0.10	0.01
Presence of meaning	23.33	7.47	25.12	6.57	-1.89	-0.25

Note. Data were missing for 26 cases for income and one case for family status. Higher self-mastery scores indicate a weaker sense of personal mastery. PCS = Physical Component Scale; MCS = Mental Component Scale.

^*^p < .05

Tables 2 and 3 display the associations between background variables, mediators, and dependent variables. However, since path analysis accounts for all associations among these variables, the results are presented with a focus on model fit and path estimates.

**Table 2:** Associations between background variables and both mediators and dependent variables

Variable	Age	Gender ^a^	Family status ^b^	Education ^c^	Income	Traumatic life events	Evacuation during war ^d^
Anxiety	− 0.03	0.25^***^	− 0.18^***^	0.07	0.01	0.27^***^	0.18^***^
Depression	− 0.05	0.19^***^	− 0.22^***^	0.05	− 0.004	0.25^***^	0.17^**^
Problem-focused coping - Self	− 0.02	0.23^***^	− 0.02	0.02	− 0.02	0.22^***^	0.16^**^
Emotion-focused coping - Self	− 0.14^**^	0.15^**^	− 0.09	0.06	− 0.05	0.15^**^	0.11^*^
Dysfunctional coping - Self	− 0.01	0.16^**^	− 0.16^**^	0.04	− 0.04	0.26^***^	0.08
Self-mastery	0.02	− 0.04	− 0.02	0.03	− 0.04	0.11^*^	0.08
Problem-focused coping - Other	− 0.05	0.20^***^	− 0.06	0.07	− 0.13^*^	0.16^**^	0.13^*^
PCS	− 0.21^***^	− 0.04	0.07	0.06	0.11^*^	− 0.32^***^	− 0.17^***^
MCS	0.13^*^	− 0.21^***^	0.13^*^	− 0.11^*^	− 0.05	− 0.22^***^	− 0.24^***^
Search for meaning	− 0.03	0.12^*^	− 0.08	0.09	− 0.03	0.02	0.10^*^
Presence of meaning	0.11^*^	0.05	0.19^***^	− 0.03	0.05	− 0.11^*^	0.32^***^

Note. *N* = 366. Higher self-mastery scores indicate a weaker sense of personal mastery. PCS = Physical Component Scale; MCS = Mental Component Scale

^a^ 0 = Male, 1 = Female; ^b^ 0 = Other, 1 = Married or in a relationship; ^c^ 0 = Non-academic, 1 = Academic; ^d^ 0 = No, 1 = Yes

^*^p < .05. ^**^p < .01. ^***^p < .001

**Table 3:** Correlations between mediators and dependent variables

Variable		M	SD	1	2	3	4	5	6	7	8	9	10
1.	Anxiety	2.28	1.79	─									
2.	Depression	2.24	1.85	0.80^***^	─								
3.	Problem-focused coping - Self	18.97	5.34	0.31^***^	0.27^***^	─							
4.	Emotion-focused coping - Self	26.00	5.80	0.32^***^	0.26^***^	0.71^***^	─						
5.	Dysfunctional coping - Self	15.46	4.15	0.45^***^	0.50^***^	0.38^***^	0.38^***^	─					
6.	Self-mastery	2.11	0.53	0.31^***^	0.33^***^	− 0.08	0.02	0.28^***^	─				
7.	Problem-focused coping - Other	17.59	6.42	0.20^***^	0.12^*^	0.66^***^	0.59^***^	0.32^***^	− 0.05	─			
8.	PCS	50.56	8.96	− 0.28^***^	− 0.28^***^	− 0.17^**^	− 0.15^**^	− 0.19^***^	− 0.19^***^	− 0.07	─		
9.	MCS	39.01	11.70	− 0.60^***^	− 0.68^***^	− 0.21^***^	− 0.23^***^	− 0.41^***^	− 0.35^***^	− 0.14^**^	0.09	─	
10.	Search for meaning	22.51	7.00	0.25^**^	0.21^***^	0.31^***^	0.26^***^	0.16^**^	0.03	0.32^***^	− 0.02	− 0.19^***^	─
11.	Presence of meaning	22.55	6.76	− 0.17^**^	− 0.28^***^	0.18^***^	0.06	− 0.22^***^	− 0.33^***^	0.19^***^	0.09	0.25^***^	0.08

Note. *N* = 366. Higher self-mastery scores indicate a weaker sense of personal mastery. PCS = Physical Component Scale; MCS = Mental Component Scale

^*^p < .05. ^**^p < .01. ^***^p < .001

**Fig. 1:**
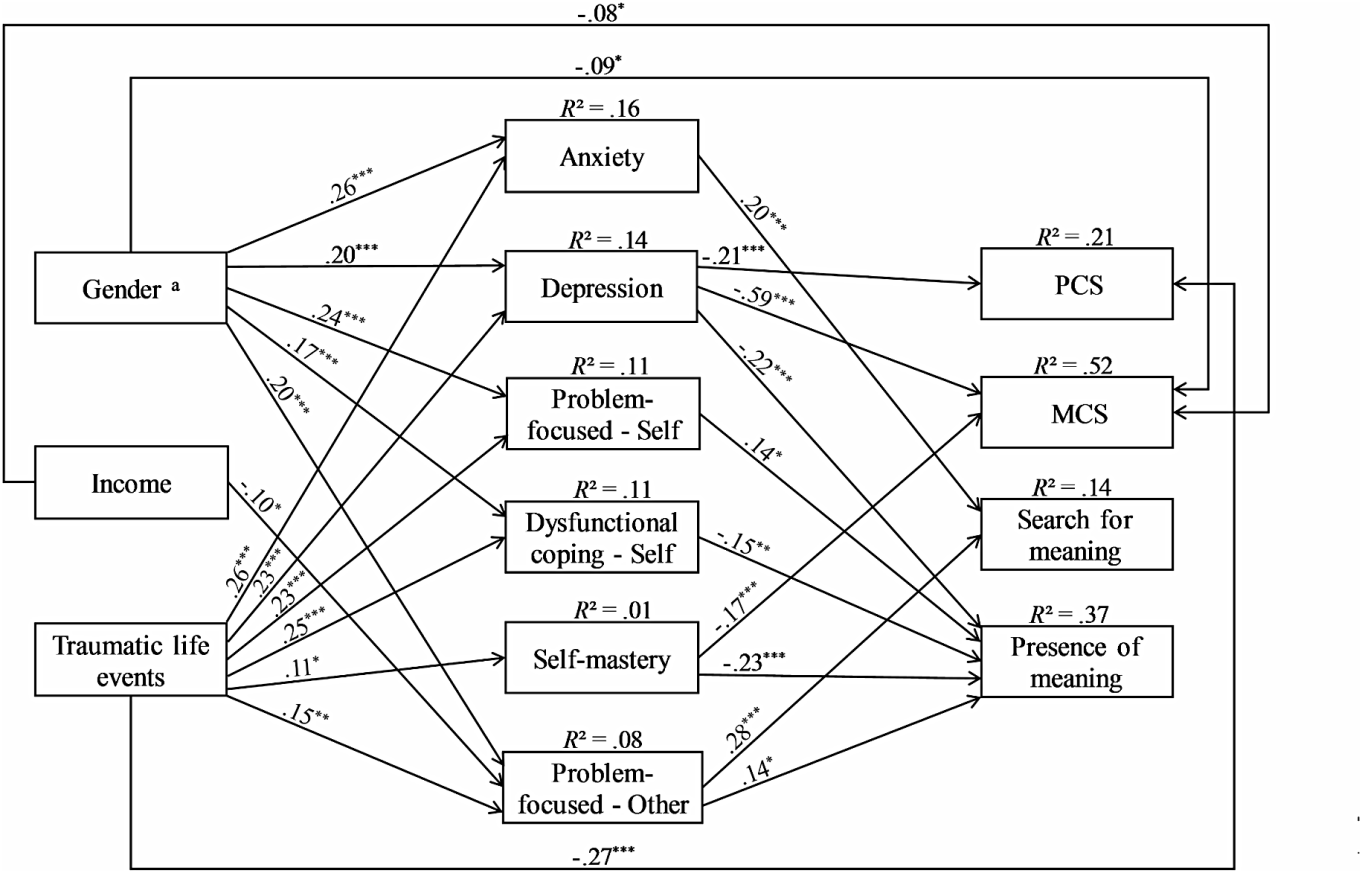
*Note. N* = 366. Standardized path coefficients are presented, controlling for age, family status, and evacuation during war. Measurement errors and correlations are not displayed for clarity (Appendix 1). Higher self mastery scores indicate a weaker sense of personal mastery. PCS = Physical Component Scale; MCS = Mental Component Scale. ^a^0 = Male, 1 = Female. ^*^p < .05. ^**^p < .01. ^***^p < .001

Following the proposed theoretical model, we conducted a path analysis to examine the hypothesized relationships among variables. The estimated model showed an excellent fit with the data. However, an examination of the path model revealed several non-significant paths, including all the paths of education and emotion-focused coping (self). Consequently, a more parsimonious model was evaluated. A comparison between the initial and parsimonious models favored the latter. Additionally, incorporating intercorrelations between the error terms of mediators and between the error terms of dependent variables further improved the model fit, making the parsimonious-modified model the most favorable. The final model, depicted in Fig. 1, exhibited an excellent fit with the data (see Table 4).

**Table 4:** Fit and model comparison indices

Model	χ^2^	df	χ^2^/df	CFI	RMSEA	SRMR	ECVI	AIC	BIC
Initial	104.50	60	1.74	0.98	0.05	0.03	1.11	404.50	989.89
Parsimonious	83.72	58	1.44	0.98	0.04	0.04	0.66	239.72	544.13
Parsimonious + MI	64.72	56	1.16	0.99	0.02	0.04	0.62	224.72	536.93

*Note. N* = 366. CFI = Comparative Fit Index; RMSEA = Root Mean Square Error of Approximation; SRMR = Standardized Root Mean Squared Residual; ECVI = Expected Cross-Validation Index; AIC = Akaike’s Information Criterion; BIC = Bayes Information Criterion; MI = Modification Indices

The analysis of path coefficients revealed several significant relationships. Income showed a negative relationship with problem-focused coping (other) and MCS. Traumatic life events were negatively correlated with PCS, and positively associated with all mediators. Anxiety was positively related to search for meaning, whereas depression exhibited negative relationships with PCS, MCS, and presence of meaning. Additionally, problem-focused coping (self) was positively related to presence of meaning, while dysfunctional coping (self) had a negative association. Self-mastery was found to have negative relationships with both MCS and presence of meaning. That it, the stronger the sense of personal mastery was, the higher these measures were. Conversely, problem-focused coping (other) was positively linked to both search for meaning and presence of meaning.

A subsequent analysis of the indirect effects revealed that all modeled indirect effects were significant, except for the effect of income on presence of meaning through problem-focused coping (other) (see Table 5). The results indicated that being female, as opposed to male, and having experienced more traumatic life events predicted higher scores on anxiety, depression, problem-focused coping (self), dysfunctional coping (self), and problem-focused coping (other). In turn, higher anxiety scores predicted higher search for meaning scores, while higher depression scores predicted lower scores on PCS, MCS, and presence of meaning. Additionally, higher scores on problem-focused coping (self) and dysfunctional coping (self) predicted lower search for meaning scores, whereas higher scores on problem-focused coping (other) predicted higher scores on both search for meaning and presence of meaning. Furthermore, a higher number of traumatic life events was linked to higher self-mastery scores (meaning lower sense of personal mastery), which subsequently predicted lower scores on MCS and presence of meaning. In terms of income, the indirect effect on search for meaning through problem-focused coping (other) was significant, with higher income predicting lower scores on problem-focused coping (other), which subsequently predicted lower search for meaning scores (see Fig. 1).

**Table 5:** Bootstrap results for the indirect effects

		95% bootstrap CI		
Indirect effect	B	Lower	Upper	β
Gender → Anxiety → Search for meaning	0.74	0.32	1.23	0.05
Gender → Depression → PCS	-0.76	-1.36	-0.31	− 0.04
Gender → Depression → MCS	-2.79	-4.14	-1.56	− 0.12
Gender → Depression → Presence of meaning	-0.60	-1.08	-0.22	− 0.04
Gender → Problem-focused coping - Self → Presence of meaning	0.48	0.06	1.01	0.03
Gender → Avoidant coping - Self → Presence of meaning	-0.34	-0.78	-0.05	− 0.02
Gender → Problem-focused coping - Other → Search for meaning	0.81	0.36	1.36	0.06
Gender → Problem-focused coping - Other → Presence of meaning	0.38	0.03	0.87	0.03
Income → Problem-focused coping - Other → Search for meaning	-0.16	-0.33	-0.01	− 0.03
Traumatic life events → Anxiety → Search for meaning	0.42	0.18	0.71	0.05
Traumatic life events → Depression → PCS	-0.50	-0.88	-0.20	− 0.05
Traumatic life events → Depression → MCS	-1.83	-2.67	-1.08	− 0.14
Traumatic life events → Depression → Presence of meaning	-0.39	-0.72	-0.15	− 0.05
Traumatic life events → Problem-focused coping - Self → Presence of meaning	0.26	0.03	0.54	0.03
Traumatic life events → Dysfunctional coping - Self → Presence of meaning	-0.29	-0.60	-0.05	− 0.04
Traumatic life events → Self-mastery → MCS	-0.24	-0.53	0.00	− 0.02
Traumatic life events → Self-mastery → Presence of meaning	-0.19	-0.43	0.00	− 0.02
Traumatic life events → Problem-focused coping - Other → Search for meaning	0.33	0.11	0.63	0.04
Traumatic life events → Problem-focused coping - Other → Presence of meaning	0.16	0.01	0.37	0.02

*Note. N* = 366. Only the significant indirect effects are shown for clarity. Higher self-mastery scores indicate a weaker sense of personal mastery. CI = Confidence Interval; PCS = Physical Component Scale; MCS = Mental Component Scale

^a^ 0 = Male, 1 = Female

Finally, the multiple group path analysis on the parsimonious modified model indicated that the unconstrained model had a better fit than the constrained model (Δχ^2^ = 57.33, Δ*df* = 32, *p* = .004). Furthermore, comparing the paths of evacuees showed several significant moderations: the association between traumatic life events and dysfunctional coping (self) was positive, while it was non-significant for those who were not evacuated. In contrast, the positive relationship between anxiety and search for meaning, as well as the negative relationship between self-mastery and MCS, were significant for participants who were not evacuated, but non-significant for those who were evacuated (see Table 6). However, all relevant moderate-mediation analyses resulted in non-significant results (all *p*’s > 0.277).

**Table 6:** Path comparisons by evacuation during war

Path	Not evacuated β	Evacuated β	Δβ	*p*_Δ_
Traumatic life events → Dysfunctional Coping - Self	0.08	0.33^***^	− 0.25	0.018
Anxiety → Search for meaning	0.32^***^	0.12	0.20	0.024
Self-mastery → MCS	− 0.31^***^	− 0.09	− 0.22	0.007

*Note. N* = 366. Only the significant differences in paths are shown for clarity. Higher self-mastery scores indicate a weaker sense of personal mastery. MCS = Mental Component Scale

^***^p < .001

For all additional analyses and supplementary information, please refer to the supplementary appendices (1–3).

## Discussion

This study, conducted in May 2024, examined evacuees from the southern and northern regions of Israel, as well as individuals who were not evacuated because they lived farther inland. All evacuees in our study were evacuated within the first month following October 7, 2023—the onset of the war. Evacuees from the northern and southern regions showed no significant differences in most study variables, except for self-mastery. The similarity in the responses of the evacuees needs to be thoroughly explained, especially given the regional variations in the intensity of military actions, with the south experiencing more severe immediate impacts. It is possible that the concept of a shared reality of displacement may explain the uniformity in traumatic experiences across regions [65]. This concept refers to the collective sense of threat, mutual understanding, and shared experiences of being displaced that arise during nationwide crises, affecting individuals regardless of their proximity to direct conflict zones. Furthermore, the war was ongoing in both the north and the south, albeit with less intensity in the north. Media coverage, national alerts, and the mobilization of reserves contributed to a pervasive atmosphere of threat throughout the country. This widespread exposure and the shared experience of displacement may have led to similar psychological impacts among evacuees from different regions, as the collective experience of war can transcend geographical boundaries [66, 67].

We examined a multivariable mediation model based on Taylor’s [3] framework to identify the mechanisms affecting HRQoL - specifically the physical component summary (PCS) and mental component summary (MCS) - and meaning in life (MIL) - including presence of meaning and search for meaning Taylor’s conceptualization provides a foundational framework for interpreting our findings, particularly in relation to aims two- four. The results concerning the entire sample revealed that traumatic life events were negatively associated with Physical Component Summary (PCS) scores and positively linked to all mediators, including the adapted subscale measuring problem-focused coping related to helping others. This indicates that individuals who experienced more traumatic events tended to struggle more with anxiety, depression, and maladaptive coping strategies across the entire sample. However, they also demonstrated resilience through adaptive strategies such as problem-focused and emotion-focused self-coping, self-mastery, and aiding others through problem-solving coping.

By expanding the model to include MIL as a dependent variable, we observed that anxiety was positively related to the search for meaning in life, highlighting that heightened anxiety can drive individuals to seek purpose. Depression was negatively associated with PCS, the Mental Component Summary (MCS), and the presence of meaning, suggesting that it hinders psychological well-being across the sample. Direct connections were also established between coping strategies and MIL. Problem-focused self-coping demonstrated a positive association with the presence of MIL, while dysfunctional coping exhibited a negative correlation. Additionally, problem-focused coping related to helping others was positively associated with both the search for meaning and the presence of meaning in life.

These findings emphasize the significance of MIL as a complementary factor in evaluating quality of life for the entire sample, highlighting its ability to provide a sense of purpose even in challenging circumstances. The pervasive atmosphere of war impacted all participants, contributing to these results. Engaging in prosocial behaviours and focusing on helping others allowed individuals who had endured traumatic events to find renewed purpose and meaning, effectively shifting attention away from their own condition [6],7. It is interesting to note that individuals with an established socioeconomic status (SES) tend to engage less in caring behavior towards others [11]. Self-coping strategies, particularly among women and those who have experienced multiple traumatic life events, are linked to increased anxiety and depression. These negative emotional states can ultimately lead to lower scores in the search for MIL. Previous findings suggest that employing adaptive coping strategies helped mitigate symptoms of distress and enhanced satisfaction with life (68–69).

In our study, we examined coping strategies related to helping others. While existing research has primarily focused on how individuals perceive social support from others, coping strategies that involve aiding others not only mitigate the effects of stress but also enable individuals to better understand and articulate the support they have provided, touching upon underexplored interpersonal aspects of coping [33, 34]. Our findings revealed that only the problem-solving subscale demonstrated reliability. This provides limited insight into how individuals employ various coping strategies to support others. To gain a deeper understanding of these dynamics, we recommend that future research incorporate comprehensive assessments of the types, frequencies, and qualities of coping strategies aimed at helping others.

By integrating both intrapsychic and interpsychic coping mechanisms, our study offers a comprehensive understanding of the multifaceted ways individuals manage stress and adversity. This approach aligns theoretically with the concept of community belonging. By engaging in altruistic behaviors, individuals potentially strengthen their social support networks and reinforce their sense of belonging [70]. Such actions not only contribute to personal coping but also enhance communal resilience, underscoring the interconnected nature of individual well-being and collective solidarity.

Concerning the fifth aim of the study, which compared the pathways between evacuees and non-evacuees, we found a positive association between traumatic life events and dysfunctional self-coping among evacuees. In contrast, these pathways were non-significant for those who were not evacuated. Dysfunctional coping is a strategy used to avoid provoking threatening feelings by steering clear of stimuli that trigger such emotions [22], aiming to reduce emotional stress. It is possible that, due to the immediate stressors and uncertainties associated with evacuation, the loss of familiar environments, daily routines, and support networks, evacuees were more inclined to employ dysfunctional coping mechanisms. In line with this notion, traumatic events illustrate “loss spirals”, a key concept of the COR theory [11]. Accordingly, evacuees, compared to non-evacuated participants, did not have a stable environment that might have allowed for a more diverse range of coping strategies, which have been found to mitigate reliance on maladaptive coping such as avoidance [71].

Interestingly, among non-evacuated participants, we observed a significant positive relationship between anxiety and the search for meaning. This suggests that individuals who remain in their homes might channel their anxiety into a constructive pursuit of personal significance or purpose. This finding aligns with Frankl’s existential perspective, which posits that the search for meaning is a fundamental human motivation, particularly in the face of adversity [7]. Furthermore, it corroborates previous research indicating that individuals confronting chronic stressors may adopt meaning-making as a coping strategy, thereby buffering against anxiety and promoting psychological well-beingbeing [75].

Finally, the negative relationship between self-mastery and MCS scores was significant among non-evacuated participants, but not among those evacuated. This suggests that among non-evacuees in a relatively stable environment, a stronger sense of self-mastery was associated with better mental health [62]. According to COR theory [11], self-mastery may serve as a personal resource that can empower individuals to develop their abilities and capacities, thereby reducing stressors and enhancing well-being [14, 72, 73].

### Limitations

This study has several limitations that warrant consideration. First, the cross-sectional design, with data collected at a single point in time, limits the ability to draw causal inferences during ongoing wartime, especially when some areas are experiencing intense fighting while others are relatively calm. The absence of pre-assessment for mental health indicators, such as anxiety and depression, further necessitates caution in data interpretation. Another limitation of our study is the way we assessed traumatic events. We used the Traumatic Life Events Questionnaire [52] and found no significant differences between the two groups of evacuees. However, it is possible that this tool was not sensitive enough to detect nuanced variations in trauma exposure. A more detailed and precise questionnaire may be necessary to accurately capture the complexity and intensity of these experiences, especially in situations of displacement caused by war. Additionally, we did not assess traumatic grief, which is characterized by the simultaneous experience of trauma and loss and has profound, long-lasting psychological impacts. Therefore, we recommend that future studies explore this important topic. Furthermore, we did not measure the impact of trauma on shifts in roles and responsibilities, nor did we assess community belonging among the study groups. Community belonging is a vital factor in understanding psychosocial outcomes, as it encompasses an individual’s sense of connection, acceptance, and identification with their community [74]. While we believe that assessing coping strategies related to helping others reflects intrinsic mechanisms of community belonging, our measurements did not explicitly include all its dimensions, such as participation in community activities, shared values, and trust within the community [75]. Engaging in altruistic behaviors can strengthen social support networks and reinforce one’s sense of belonging within the community [70]. However, we acknowledge that these unmeasured factors could have provided additional insights into psychosocial outcomes. Future studies should incorporate specific measures of community belonging and support to expand upon our findings.

We also acknowledge the need for comprehensive evaluations of the types, frequencies, and qualities of therapeutic interventions and other support systems available to the evacuees. Future investigations could examine how varying levels of therapy influence the development of coping strategies, psychological resilience, and overall well-being. Finally, reliance on self-report data may introduce cognitive biases and social desirability effects. To address this, future research should incorporate multiple data sources and objective measures where possible. Moreover, the generalizability of our study is limited due to its focus on an Israeli population with unique cultural characteristics. To enhance external validity, future research should aim to replicate these findings across diverse cultural contexts and populations.

### Implication for health policy

Grounded by the theoretical frameworks of the COR theory [11], Taylor’s “tend and befriend” model [32], and Frankl’s existential framework [76], our study emphasizes the necessity for comprehensive health care policies that holistically address the needs of individuals affected by conflict-induced evacuation. Specifically, health care systems should prioritize the integration of mental and physical health services to provide a unified approach to treatment. Interdisciplinary collaboration is imperative for adapting policies to evolving needs and ensuring comprehensive care.

Additionally, implementing trauma-informed care practices is crucial; health care providers must be trained to recognize and sensitively respond to the effects of trauma, including evacuation itself, thereby creating stable and supportive environments [77]. Likewise, incorporating meaning-centered therapeutic interventions inspired by Frankl’s existential perspective can facilitate the search for purpose, which is particularly healing in times of profound distress. Targeted interventions should prioritize enhancing adaptive coping strategies (problem-focused coping) while addressing the impacts of income and traumatic experiences on mental health. Thus, mental health professionals should focus on strengthening these adaptive coping mechanisms to mitigate the negative psychological impacts of trauma. Notably, the study indicates that education and emotion-focused coping may play a less critical role than initially thought, potentially redirecting future research efforts.

By fostering opportunities for individuals to engage in altruistic activities—aligned with Taylor’s Tend-and-Befriend model and Frankl’s ideas—health care policies can enhance coping mechanisms that involve social support and community engagement. This approach not only aids individual recovery but also strengthens social cohesion within displaced communities. Additionally, addressing resource loss, as highlighted by COR theory, should be a critical component of health care strategies. By integrating social services that assist with housing, employment, and financial stability within health care settings, providers can alleviate external stressors that exacerbate health issues.

Culturally sensitive and individualized care must also be emphasized to meet the diverse needs of evacuees, ensuring that interventions are respectful and effective across different cultural contexts. Enhancing accessibility to health care through mobile clinics, telemedicine, and multilingual services will further reduce barriers faced by evacuees.

## Conclusions

The research highlights the need for trauma-informed care that acknowledges the complex impacts of traumatic life events on HRQOL. This study advocates for a paradigm shift in social policy towards a comprehensive understanding of well-being—one that considers not only physical and mental health, but also the crucial role of meaning-making and prosocial coping in achieving resilience, especially during wartime and traumatic experiences.

## Electronic supplementary material

Below is the link to the electronic supplementary material.

Supplementary Material 1

## Data Availability

The datasets of the current study are available from the corresponding author upon reasonable request.

## Abbreviations

HRQOL: Health-related quality of life
MIL: Meaning in life
SES: Socioeconomic status
PCS: Physical Component Summary
MCS: Mental Component Summary
COR: Conservation of resources
