# Supplementary material for: Coping, meaning in life, and quality of life during ongoing conflict: insights from Israeli populations
Source: Isr J Health Policy Res. 2025 Jan 8;14:1. doi: 10.1186/s13584-024-00665-1 (PMC11715607; doi:10.1186/s13584-024-00665-1)
Supplement: Supplementary file 1 — Supplementary Material 1 [file 13584_2024_665_MOESM1_ESM.docx]

**Appendix 1.** Means and standard deviations of mediators and dependent variables by dichotomous background variables

| **Variable** | **Gender** | | **Family status** | | **Education** | | **Evacuation during war** | |
| --- | --- | --- | --- | --- | --- | --- | --- | --- |
|  | **Male** | **Female** | **Married or in a relationship** | **Other** | **Not academic** | **Academic** | **No** | **Yes** |
|  | **(*n =* 136)** | **(*n =* 230)** | **(*n =* 222)** | **(*n =* 144)** | **(*n =* 179)** | **(*n =* 187)** | **(*n =* 145)** | **(*n =* 221)** |
| Anxiety | 1.70 | 2.62 | 2.01 | 2.68 | 2.15 | 2.40 | 1.88 | 2.54 |
|  | 1.52 | 1.86 | 1.65 | 1.93 | 1.81 | 1.77 | 1.65 | 1.84 |
| Depression | 1.79 | 2.51 | 1.91 | 2.75 | 2.14 | 2.34 | 1.86 | 2.50 |
|  | 1.73 | 1.86 | 1.75 | 1.88 | 1.76 | 1.93 | 1.69 | 1.90 |
| Problem-focused coping - Self | 17.38 | 19.90 | 18.90 | 19.08 | 18.85 | 19.08 | 17.93 | 19.65 |
|  | 5.08 | 5.28 | 5.21 | 5.55 | 5.45 | 5.25 | 5.00 | 5.46 |
| Emotion-focused coping - Self | 24.89 | 26.66 | 25.58 | 26.65 | 25.68 | 26.32 | 25.23 | 26.51 |
|  | 5.77 | 5.73 | 5.75 | 5.84 | 5.57 | 6.01 | 5.42 | 6.00 |
| Dysfunctional coping - Self | 14.62 | 15.96 | 14.93 | 16.28 | 15.27 | 15.64 | 15.04 | 15.73 |
|  | 4.50 | 3.85 | 3.85 | 4.46 | 4.00 | 4.30 | 3.89 | 4.30 |
| Self-mastery | 2.14 | 2.09 | 2.10 | 2.12 | 2.09 | 2.12 | 2.06 | 2.14 |
|  | 0.58 | 0.49 | 0.49 | 0.58 | 0.51 | 0.54 | 0.49 | 0.55 |
| Problem-focused coping - Other | 15.88 | 18.60 | 17.26 | 18.09 | 17.13 | 18.02 | 16.60 | 18.24 |
|  | 6.29 | 6.30 | 6.56 | 6.20 | 6.26 | 6.57 | 6.28 | 6.45 |
| PCS | 50.97 | 50.32 | 51.08 | 49.75 | 49.98 | 51.12 | 52.45 | 49.32 |
|  | 8.10 | 9.44 | 8.58 | 9.48 | 8.91 | 8.99 | 7.43 | 9.65 |
| MCS | 42.26 | 37.09 | 40.21 | 37.16 | 40.36 | 37.72 | 42.52 | 36.71 |
|  | 11.82 | 11.22 | 11.24 | 12.18 | 11.09 | 12.14 | 10.61 | 11.83 |
| Search for meaning | 21.38 | 23.17 | 22.09 | 23.16 | 21.87 | 23.12 | 21.61 | 23.10 |
|  | 6.77 | 7.07 | 6.71 | 7.42 | 7.10 | 6.88 | 7.09 | 6.90 |
| Presence of meaning | 22.13 | 22.80 | 23.59 | 20.94 | 22.75 | 22.35 | 19.88 | 24.29 |
|  | 7.05 | 6.58 | 6.33 | 7.09 | 6.66 | 6.86 | 5.31 | 7.04 |
| *Note.* Each cell represents means (above) and standard deviations (below). PCS = Physical Component Scale; MCS = Mental Component Scale | | | | | | | | |

**Appendix 2.** Intercorrelations of study variables

| **Variable** | **Correlation** | ***p*** |
| --- | --- | --- |
| *Independent* |  |  |
| Age ↔ Gender ^a^ | -.01 | .866 |
| Age ↔ Family status ^b^ | .16 | .003 |
| Age ↔ Income | .10 | .061 |
| Age ↔ Traumatic life events | .02 | .676 |
| Age ↔ Evacuation during war ^c^ | .01 | .891 |
| Gender ^a^ ↔ Family status ^b^ | -.08 | .151 |
| Gender ^a^ ↔ Income | -.08 | .115 |
| Gender ^a^ ↔ Traumatic life events | -.04 | .509 |
| Gender ^a^ ↔ Evacuation during war ^c^ | .18 | .001 |
| Family status ^b^ ↔ Income | .20 | < .001 |
| Family status ^b^ ↔ Traumatic life events | -.13 | .016 |
| Family status ^b^ ↔ Evacuation during war ^c^ | .02 | .670 |
| Income ↔ Traumatic life events | -.21 | < .001 |
| Income ↔ Evacuation during war ^c^ | -.08 | .151 |
| Traumatic life events ↔ Evacuation during war ^c^ | .13 | .014 |
| *Mediators* |  |  |
| Anxiety ↔ Depression | .77 | < .001 |
| Anxiety ↔ Problem-focused coping - Self | .22 | < .001 |
| Anxiety ↔ Dysfunctional coping - Self | .37 | < .001 |
| Anxiety ↔ Self-mastery | .34 | < .001 |
| Anxiety ↔ Problem-focused coping - Other | .09 | .009 |
| Depression ↔ Problem-focused coping - Self | .19 | < .001 |
| Depression ↔ Dysfunctional coping - Self | .42 | < .001 |
| Depression ↔ Self-mastery | .35 | < .001 |
| Problem-focused coping - Self ↔ Dysfunctional coping - Self | .34 | < .001 |
| Problem-focused coping - Self ↔ Problem-focused coping - Other | .63 | < .001 |
| Dysfunctional coping - Self ↔ Self-mastery | .31 | < .001 |
| Dysfunctional coping - Self ↔ Problem-focused coping - Other | .26 | < .001 |
| *Dependent variables* |  |  |
| PCS ↔ MCS | -.19 | < .001 |
| MCS ↔ Presence of meaning | .14 | .006 |
| *Note*. *N* = 366. PCS = Physical Component Scale; MCS = Mental Component Scale | | |
| ^a^ 0 = Male, 1 = Female; ^b^ 0 = Other, 1 = Married; ^c^ 0 = No, 1 = Yes | | |

**Appendix 3.** Paths from covariates to mediators and dependent variables

| **Path** | **β** | ***p*** |
| --- | --- | --- |
| Age → PCS | -.21 | < .001 |
| Age → MCS | .12 | .002 |
| Age → Presence of meaning | .09 | .029 |
| Family status ^a^ → Anxiety | -.14 | < .001 |
| Family status ^a^ → Depression | -.19 | < .001 |
| Family status ^a^ → Dysfunctional coping - Self | -.12 | .006 |
| Family status ^a^ → Presence of meaning | .11 | .014 |
| Evacuation during war ^b^ → PCS | -.10 | .034 |
| Evacuation during war ^b^ → MCS | -.12 | < .001 |
| Evacuation during war ^b^ → Presence of meaning | .34 | < .001 |
| *Note*. *N* = 366. PCS = Physical Component Scale; MCS = Mental Component Scale | | |
| ^a^ 0 = Other, 1 = Married or in a relationship; ^b^ 0 = No, 1 = Yes | | |
